# The need for better donor engagement and outreach with legacy living kidney donors

**DOI:** 10.1093/ckj/sfad271

**Published:** 2023-10-19

**Authors:** Katya Loban, Ahsan Alam, Shaifali Sandal

**Affiliations:** Division of Nephrology, Department of Medicine, McGill University Health Centre, Montreal, Quebec; Research Institute of the McGill University Health Centre, Montreal, Quebec; Division of Nephrology, Department of Medicine, McGill University Health Centre, Montreal, Quebec; Research Institute of the McGill University Health Centre, Montreal, Quebec; Division of Experimental Medicine, Department of Medicine, McGill University Health Centre, Montreal, Quebec; Division of Nephrology, Department of Medicine, McGill University Health Centre, Montreal, Quebec; Research Institute of the McGill University Health Centre, Montreal, Quebec; Division of Experimental Medicine, Department of Medicine, McGill University Health Centre, Montreal, Quebec

To the Editor,

Until a decade ago, living kidney donors (LKDs) were not perceived as ‘patients’ needing ongoing medical attention, but rather as members of the general population who have recovered from surgery [1]. This was likely because older literature reported minimal risk of kidney donation when compared with an unscreened general population [2]. However, a growing body of evidence now reports quantifiable long-term risks of kidney donation [2, 3]. Thus, there is now a concerted effort to improve post-donation care and follow-up, such as in the US, where transplant programs have been required to submit follow-up data since 2005 and in 2013 specific thresholds for data submission and completeness were described. We want to highlight the case of a LKD and argue that this is not enough, particularly for ‘*legacy*’ LKDs, i.e. those who donated a kidney decades ago.

Through an 86-year-old kidney transplant recipient, we discovered that his son and LKD, now 53 years of age, developed a left hemispheric intracerebral hemorrhage due to uncontrolled hypertension. The son had donated a kidney 17 years prior at the age of 36 when he had no relevant medical history. Post-donation his creatinine was in the 1–1.3 mg/dL range and he had no albuminuria. Similar to other centers, we defer the follow-up of LKDs to a family doctor [4]. However, he lost his family doctor to retirement and after that, the LKD felt he was not aggressive in pursuing one as he perceived himself to be healthy and that is what he was told when he donated. A year since this event, he had several right-sided deficits and his blood pressure was controlled on four anti-hypertensive agents (including a renin-angiotensin-aldosterone system inhibitor), but his renal function was stable.

Transplant programs are privy to emerging literature, and the consent and education process of new LKD candidates incorporates the most up-to-date evidence. However, how and if this information is communicated to prior LKDs who donated when these risk assessments were informed by older data is unknown. Early detection of modifiable risk factors for serious cardiovascular and renal complications can inform timely interventions and preventative measures. Our LKD was at a higher risk of hypertension than can be estimated with normal aging [5]. This literature emerged after he donated and communicating this risk to him could have potentially changed his clinical course.

Currently, few channels of communication exist between donor programs and LKDs beyond the first few months [4]. We do not advocate that transplant programs follow LKDs long-term given they may lack the resources and infrastructure to do so. In addition, care rendered by them may be more costly to healthcare systems. Given the continuously evolving literature and better characterization of risks associated with donation, we instead recommend creating mechanisms of better engagement and outreach with LKDs over their entire lifetime that keep them abreast of emerging literature on the risks of living kidney donation and remind them to seek regular follow-up with their primary doctor. Transplant programs can send newsletters and emails, create social media posts, arrange seminars or pursue other telehealth approaches with prior LKDs on a periodic basis. The corresponding transplant recipient can also be engaged to support their LKD.

Lastly, given that LKD care is frequently provided at the community level, we also advocate for an integrated model of comprehensive care across the primary and transplant care continuum. This may facilitate the communication of recent and robust data to primary care practitioners and ensure evidence-based practices are followed in donor care [4]. LKDs have helped significantly advance the field of kidney transplantation, thus, it is the responsibility of the medical community to ensure that the care of ‘*legacy*’ LKDs is also prioritized.

Consent to publish this case was obtained from both the living kidney donor and the recipient.

## References

[1] Leichtman A , Abecassis M, Barr M et al. Living kidney donor follow-up: state-of-the-art and future directions, conference summary and recommendations. Am J Transplant 2011;11:2561–8. 10.1111/j.1600-6143.2011.03816.x 22054039

[2] Lentine KL , Lam NN, Segev DL. Risks of living kidney donation: current state of knowledge on outcomes important to donors. Clin J Am Soc Nephrol 2019;14:597–608. 10.2215/CJN.11220918 30858158 PMC6450354

[3] Lentine KL , Kasiske BL, Levey AS et al. KDIGO clinical practice guideline on the evaluation and care of living kidney donors. Transplantation 2017;101:S7–S105. 10.1097/TP.0000000000001769 PMC5540357 28742762

[4] Loban K , Robert JT, Alam A et al. Long-term care of living kidney donors needs a better model of healthcare delivery. Prog Transplant 2023;33:242–6. 10.1177/15269248231189879 PMC10466930 37475465

[5] Boudville N , Prasad GV, Knoll G et al. Meta-analysis: risk for hypertension in living kidney donors. Ann Intern Med 2006;145:185–96. 10.7326/0003-4819-145-3-200608010-00006 16880460

